# Biotransformation of Progesterone by Whole Cells of Filamentous Fungi *Aspergillus brasiliensis*

**Published:** 2015

**Authors:** Tahereh Hosseinabadi, Hossein Vahidi, Bahman Nickavar, Farzad Kobarfard

**Affiliations:** a *Department of Pharmacognosy, School of Pharmacy, Shahid Beheshti University of Medical Sciences, Tehran, Iran.*; b *Department of Pharmaceutical Biotechnology, School of Pharmacy, Shahid Beheshti University of Medical Sciences, Tehran, Iran.*; c *Department of Medicinal Chemistry, School of Pharmacy, Shahid Beheshti University of Medical Sciences, Tehran, Iran.*; d *Phytochemistry Research Center, Shahid Beheshti University of Medical Sciences, Tehran, Iran.*

**Keywords:** Progesterone, *Aspergillus brasiliensis*, Microbial transformation, Steroid, Hydroxylation

## Abstract

Microbial steroid biotransformations have found a wide-reaching application for the production of more precious and functionalized compounds due to their high regio-and stereo selectivity.

In this study, the possibility of using filamentous fungi *Aspergillus brasiliensis*cells in the biotransformation of progesterone, a C-21 steroid hormone was studied for the first time.

The fungal strain was inoculated into the transformation medium supplemented with progesterone as a substrate. Biotransformation of this steroid for 7 days afforded 3 different hydroxylated metabolites: 11*α*-hydroxy progesterone; 14*α*-hydroxyprogesteroneand21-hydroxyprogesterone.

The metabolites were separated by thin layer chromatography. Structure determinations of the metabolites were performed by comparing NMR, MS and IR spectra of the starting compound with those of metabolites.

These results may be of industrial importance because the metabolites can be used as precursor of some steroid drugs.

## Introduction

Steroid drugs are synthesized by chemical or microbial routes, both of which involve conversion of steroid precursors to drug intermediates and final conversion of intermediates to active drugs (1, 2).

Microorganisms have been widely employed for (different productsbioconversion and specially) steroids biotransformation to prepare derivatives, the production of which is difficult to obtain by other methods (3, 4).

The value of microbial biotechnology in the production of steroid drugs was realized for the first time in 1952 when the process of 11*α*-hydroxylation of progesterone by *Rhizopus* species was patented by Murray and Peterson (5). Fungi are widely used in steroid microbial transformation studies, since variety of their multipurpose enzyme sallow them to transform anextensive range of steroids (6) .

Thus far, different *Aspergillus* species have been used for the biotransformation of many steroids. These biotransformation evidenced interesting results, such as Baeyer -Villiger oxidations, 5*α*-reductions and different microbial hydroxylations on steroid skeleton (6, 7).

There are some reports of biotransformation of progesterone by many strains of *aspergillus* like *A. wentti, A. niger, A. nidulans, A. ochraceus, A. parasiticus, A. oryzae*، *A. flavu*s, *A. tamari, A. parasiticus *and* A. fumigatus *(6, 8-10). 

*Aspergillus brasiliensis* which is described within *Aspergillus* section *Nigri* has very good growth and sporulation. This species can be distinguished from other black aspergilli based on intergenic transcribed region, beta-tubulin and calmodulin gene sequences, by amplified fragment length polymorphism analysis and by extrolite profiles (11, 12). 

As far as biotransformations by *A. brasiliensis* are concerned, we have not found any report indicating the biotransformation of steroids by this microorganism. Actually, microbial transformations by this fungus on any substarate have not been reported in literature. The aim of present study was to explore the ability of *A. brasiliensis *in biotransformation of progesterone, as an important steroid for the first time.

## Experimental

*Materials*

Progesterone was purchased from Sigma-Aldrich company and used as substrate for biotransformation. 

All other chemicals and reagents were of analytical grade and commercially available.

*Instrumental methods*

The Infrared spectra (IR) were obtained on a Perkin-Elmer 843 spectrometer with KBr as adiluent. Electrospray ionization mass spectra (ESI-MS) were obtained using Agilent 6410 Triple Quad mass spectrometer. The ^1^H and ^13^C nuclear magnetic resonance (NMR) spectra were obtained using a Bruker DRX (Avance 500) spectrometer (Rheinstetten, Germany) at 500 and 125 MHz, respectively, with tetramethyl silane (TMS) as internal standard in CDCl_3_. Chemical shifts (δ) are given in parts per million (ppm) relative to TMS. Coupling constants (J) are given in hertz (Hz). Thin layer chromatography (TLC) was performed, on 0.25 thick layers of silica gel G (Kiselgel 60 HF_254+366_, Merck). Chromatography was performed with chloroform/acetone (7:3) and visualized by spraying the plates with a mixture of methanol–sulfuric acid (6:1) and heating in an oven at 100 ^◦^C for 3 min until the colors developed. Melting points(mp) were measured by thermo scientific 9200 apparatus and were uncorrected.

*Microorganism*

The fungal strain *A. brasilliensis *PTCC 5298 used in the present work was purchased From Iranian Research Organization for Science and Technology (IROST) in lyophilized powder form.

Cultures of fungi were grown at 26 °C for 5 days until good sporulation was obtained on Czapek. Stock cultures were maintained at 4 °C on Czapek medium slopes and freshly subcultured before use in transformation experiments. The organism was transferred to fresh medium and refreshed every two weeks. 

*Inoculum preparation and biotransformation process*

Five day old spores were washed from slants with distilled water containing Tween-80 and aseptically inoculated in 500 mL Erlenmeyer flask containing 100 mL of the growth medium under aseptic condition (pH of the medium was adjusted to 7.4 before sterilization). 

Volume of inoculums, containing 1 x 10^6^ spores, was used in all experiments unless otherwise stated. The used biotransformation medium was the same as that of growth medium. The flasks were incubated on a rotary shaker at 125 rpm, 26 ± 1 °C for 48 h until pellet formation. 

100 mg of progesterone dissolved in 1 mL acetone was added to a 48 h-grown culture and incubated to continue the transformation. A parallel control that received no progesterone and also a culture medium without microorganism, containing the substrate were ran concurrently (Control cultures).

Biotransformation was carried out under above condition for further 7 days(13).

Sampling was carried out every 24 h. The samples (5cc) were extracted with three volumes of chloroform and analyzed by thin layer chromatography (TLC).

*Product isolation and analyses*

At the end of incubation after the transformation, both mycelia and filtrate were separately extracted with chloroform (3 volumes), the chloroform extracts were dried over anhydrous sodium sulfate and evaporated under reduced pressure. Complete evaporation of the solvent gave a semi-solid mass from the extract of the filtrate. The residue was then loaded on TLC plates and fractionated with chloroform/ acetone (7:3, v/v) solvent system and the metabolites were separated from silica gel by a mixture of methanol/chloroform/acetone (three times). The fractions thus obtained were further purified by further chromatography. Purified metabolites were identified by the spectral data (^13^C NMR, ^1^H NMR, FTIR and MS).

## Result and Discussion

Incubation of progesterone by *A. brasiliensis* for 7 days afforded 3 main products II-IV.

**Figure 1:**
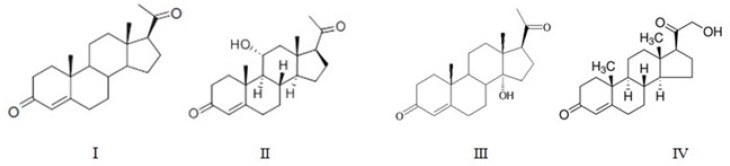
The Structure of progesterone and its metabolites

 Products of progesterone (I) bioconversion were recovered from TLC plates and their chemical structures were determined mainly based on ^1^H-NMR (Table I) and ^13^C-NMR (Table II) spectra, together with data obtained from mass spectral and FTIR spectral data. Further support for the identification of the compounds was obtained by comparison of the reported spectral values for the compounds. The molecular structures of metabolites are shown in Figure 1. 

R_f_ of progesterone and metabolites in chloroform: acetone (7:3) were 0.9, 0.53, 0.74, 0.8 respectively.

**Table 1:** 1H NMR data for compounds 1-4.

**Hydrogen atom**	**I**	**II**	**III**	**IV**
4-H	5.73	5.72	5.72	5.74
17α-H	2.53 (1H, t)	2.6(1H, t)	3.2(1H, t)	2.9(1H, t)
18-CH3	0.67	0.68(3H, s)	0.78(3H, s)	0.69(3H, s)
19-CH3	1.2	1.31	1.22	1.18
21-CH3	2.13(3H, s)	2.12 (3H, s)	2.18(3H, s)	4.21 (dd, 21*α*-H)
Other significant H signal	-	4.03 (1H, dd, 11*β*-H)	3.23 (t, 12*α*-H)	-

**Table 2:** ^13^C-NMRdata determined in CDCl3 at 500 MHz for compounds 1-4.

**Carbon atom**	**I**	**II**	**III**	**IV**
1	35.46	33.58	35.72	35.68
2	33.72	34.17	33.92	33.89
3	199.18	200.36	199.93	199.58
4	123.66	124.51	123.95	123.98
5	170.8	171.14	170.55	170.87
6	32.55	33.56	32.57	32.72
7	31.65	31.51	31.92	31.85
8	35.28	37.46	35.72	35.52
9	53.38	55.32	46.28	53.54
10	38.34	39.93	38.26	38.35
11	20.78	68.79	21.32	20.90
12	38.40	50.35	38.61	38.55
13	43.68	44.13	46.28	44.66
14	55.76	55.32	85.15	56.60
15	24.13	24.22	24.73	24.46
16	22.57	22.90	22.69	22.69
17	63.23	63.11	59.42	63.67
18	13.11	14.48	17.23	13.45
19	17.13	18.27	17.02	17.35
20	209.08	208.93	210.45	210.17
22	31.29	31.38	31.47	59.02

The mass fragmentation profile of the products revealed molecular ion peaks at m/z 330 for all the compounds II-IV, which is at 16 mass units higher than that of the parent compound, progesterone, indicating the possible insertion of one oxygen atom in the structure of each of the products.

In 

Compound II: 11α-hydroxy progesterone

The purified material had m.p. of 165-167 °C. Its IR spectrum showed an absorption bands at 3439 cm^-1^(OH) and 1699-1660 cm^-1^(conjugated CO).

The ^1^H NMR spectrum of the metabolite II demonstrated a downfield shift (δ 0.08 ppm) for the 19-methyl group. The metabolite had a characteristic resonance at δ_H_4.07 ppm due to the signal of 11*β* proton, in the shape of a triplet splitted into doublets(1H, td, J = 10.0 and 5.0 Hz) (14) and absence of the characteristic C-11 at δ 20.78 in ^13^C NMR spectrum replaced with δ_C_68.54 ppm suggesting that hydroxylation had taken place at the equatorial proton at C-11, generating an 11*α*-hydroxy group. The comparison of the metabolite’s melting point and spectral data with those in the literature (15, 16) confirmed that it was 11*α*-hydroxyprogesterone.

Compound III: 14*α*-hydroxy progesterone

This compound had m.p. of 195-197 °C. Increased polarity of the metabolite III compared to progesterone, and its IR and mass data suggested the insertion of an oxygen atom as tertiary hydroxyl functional group in the molecular structure. The infrared analysis of compound III showed two carbonyl absorption bands at 1693 and 1644 cm^-1 ^and a hydroxyl group at 3474 cm^-1^. Among the 8*β*-, 9*α*-, 14*α*- and 17*α*-tertiary positions available in progesterone, hydroxylation at 8 *β*-position should furnish a triplet for 9 *α*-proton while that at 9 *α*-position would cause a down-field shift of 19-CHз by ca. 0.13 ppm and small but significant shift of 4-H signal to ca. δ 5.9 without affecting the 17-H signal. In fact, no such situation was observed with the spectral profile of III. In contrast, a distinctive shift of 0.71 ppm (ca. δ 2.5 to 3.2) for 17*α*-H signal without any change in the triplet, an appreciable down-field shift (by 0.13 ppm) for 18-CHз but no change for 19-CHз signal are characteristic features of 14*α*-hydroxylation (13, 17).

Compound IV: 21-hydroxyprogesterone (11-Deoxycorticosterone)

Melting point of this product was142-144 °C. The IR spectrum showed two carbonyl absorption bands at 1691 and 1662 cm^-1 ^and a hydroxyl group at 3479 cm^-1^.

In case of metabolite IV, absence of characteristic C-21 at δ 31.29 in ^13^CNMR spectrum proposes the substitution of hydroxyl group in C -21 which appeared at δ 59.2. The ^1^H NMR spectrum demonstrated a large downfield shift (Δδ: 2.07 ppm) for the 21-methyl group. The characteristic resonances at δ_H_ 4.20 ppm the shape of a double of doublet, also a downfield shift of 17α-methyl protons singlet (Δδ = 0.4 ppm) without any change in the triplet, and no significant changes for 18-CHз and 19-CHз signals confirmed this metabolite as 21-hydroxyprogesterone. 

Thus far the important biotrans formations which have been reported for progesterone were 11*β*, 16*α*, 17*α* and 21*α* hydroxylation for producing corticosteroids (18, 19). Steroidal hydroxylase system of filamentous fungi is usually presented by mono oxygenase of microsomal localization which contains cytochrome P-450 (Cyt P-450) as a terminal oxidase. The cytochrome P-450 binds a substrate and flavoprotein NADP(H)-P450-reductase that provides an electron transport from reduced coenzyme to Cyt P-450 enzyme (20, 21).

The results of present study indicate that *A. brasiliensis* hydroxylated progesteronein position C_11_ and C_14 _which have been previously reported in some microbial transformations(3, 17, 22, 23). The ability of different species of *Aspergillus* such as *A. fumigatus, A. niger, A. fischerz* and *A. wentii* in bioconversion of progesterone to 11α- hydroxyl progesterone has already been reported in literature (15, 24-26). 11*α*-hydroxylation is an essential step in corticosteroid synthesis. Thus, the ability of *A. brasiliendsis *to convert I to II exemplifies the potential of this fungi for this specific purpose. In addition, 14α-hydroxylation is an important process since its product possesses anticancer properties (6). Consequently, the 14a-hydroxylation capacity of *A. brasiliendsis* may be of commercial importance.

Hydroxylation at C-21 position of the steroid skeleton was previously recorded in literature (3, 27). This substitution is an important structural feature for the biological activity of the adrenal cortical hormones (28). 21-hydroxyprogesterone or 11-Deoxycorticosterone, is a steroid hormone produced by the adrenal gland that possesses mineralocorticoid activity and acts as a precursor for aldosterone. 

## Conclusion

The results obtained in this study indicate that *A. brasiliendsis*is capable of hydroxylating the different sites of progesterone. It produces, in acceptable yields, three valuable steroidal transformation products, 11*α*-hydroxy progesterone as a major compound, 14*α*-hydroxy progesterone and 21-hydroxyprogesterone or deoxycorticosterone. To the best of our knowledge, there have been no reports of steroid biotransformation by *A. brasiliendsis* in the literature. Therefore understanding the process of biotransformation by this microorganism could be of applied importance where fungi are used for production of valuable steroids with different pharmacological properties.
